# Perspectives of young people who access support for mental health in primary care: a systematic review of their experiences and needs

**DOI:** 10.3399/BJGP.2021.0335

**Published:** 2022-02-08

**Authors:** Rebecca Appleton, Julia Gauly, Faraz Mughal, Swaran P Singh, Helena Tuomainen

**Affiliations:** Mental Health Policy Research Unit, Division of Psychiatry, UCL, London.; Warwick Medical School, University of Warwick, Coventry.; School of Medicine, Keele University, Keele; affiliate, NIHR Greater Manchester Patient Safety Translational Research Centre, Keele University, Keele; and honorary clinical research fellow, Unit of Academic Primary Care, University of Warwick, Coventry.; Warwick Medical School, University of Warwick, Coventry.; Warwick Medical School, University of Warwick, Coventry.

**Keywords:** general practice, mental health, primary health care, systematic review, young people

## Abstract

**Background:**

There is an increasing demand for mental health support in primary care, especially for young people. To improve mental health support for young people in general practice, the needs of young people must be considered.

**Aim:**

To explore the experiences of young people (aged 12–25 years) on receiving mental health care in primary care and identify the needs of young people who present with mental health concerns.

**Design and setting:**

A systematic review and narrative synthesis.

**Method:**

This was a systematic review and narrative synthesis. Six databases were searched for literature relating to young people’s experiences of receiving mental health care in primary care. Additional handsearching and manual internet searching were conducted. Narrative synthesis was employed.

**Results:**

Five papers and a further two reports from manual internet searching were found, resulting in the inclusion of 1823 young people from four different countries (UK, US, Ireland, and Canada) for synthesis. The synthesis generated four themes: the centrality of a trusting relationship; showing empathy and taking concerns seriously; being given time to talk; and barriers to accessing mental health support in primary care.

**Conclusion:**

Young people need a trusting relationship to discuss sensitive issues. To enable high-quality and effective mental health consultations with young people and the development of trust, GPs require unhurried consultations and the ability to maintain continuity of care.

## INTRODUCTION

The King’s Fund, a leading independent think tank focusing on health and care services and patient experience in England, has identified children and young people as one of the groups whose mental health needs are not currently being adequately met in primary care.^1^ GPs usually manage young people’s mental health without the involvement of specialist mental health professionals because of overstretched child and adolescent mental health services (CAMHS) and variable, fragmented, and often difficult to navigate care pathways into CAMHS,^2^ now sometimes referred to as children and young people’s mental health services, CYPHMS. The age cut-off of these services is usually 18 years, but can be as low as 16 years, even though adult mental health services (AMHS), do not accept patients until they are 18 years old. GPs across the UK have experienced an increase in the number of young people seeking support for their mental health.^3^ Late adolescence is the time when most severe mental illnesses emerge, for example, psychotic disorders.^4^ Diagnosis and the management of mental illness in young people can be challenging for GPs, more so without the involvement of mental health specialists.^5^

Mental health care provision in primary care is particularly difficult for patients with comorbidities or complex needs.^1^^,^^2^^,^^6^ Many young people who reach the boundary between child and adult mental health services fit this category. Most, however, do not meet the strict eligibility criteria for adult services,^7^ and therefore are discharged to their GP.^8^ GPs may feel ill-equipped to provide the right care for these young people,^9^ for example, prescribing certain types of psychotropic medication without input from specialist care.^10^

As mental health care from GPs is often the only available health service for young people it is important that the experiences and needs of young people are identified to improve care for young people with mental health problems in primary care. This systematic review aimed to:

- identify the experiences and views of young people (aged 12–25 years) with or without a mental health diagnosis or prior mental health service experience receiving mental health care in primary care; and
- elicit the needs of young people when visiting primary care for mental health concerns.

## METHOD

The protocol for this systematic review was registered with PROSPERO (CRD42020192845) and is reported according to PRISMA guidelines.^11^

**Table:** How this fits in

There is increasing demand for primary care-based mental health support for young people, especially for those with prior mental health service use experience. This systematic review explored young people’s experiences of support for their mental health in primary care and identified facilitators and barriers for accessing mental health care. Four themes were generated: the centrality of a trusting relationship; showing empathy and taking concerns seriously; being given time to talk; and barriers to accessing mental health support in primary care. New funding within primary care networks enables the employment of mental health practitioners based in general practices. This is an opportunity to increase mental health provision for young people in primary care.

### Search strategy

A search strategy was developed in collaboration with a specialist librarian and included search terms relating to general practice, young people, mental health, and experiences of services and was refined following scoping searches (Medline search strategy, see Supplementary Table S1). To ensure no relevant reference is missed, six bibliographic databases were searched: Embase, Medline, PsycINFO, Web of Science, ASSIA, and CINAHL from their inception to June 2020. Medical Subject Headings (MeSH) and title, abstract, and keyword screening was conducted.

The reference lists of included studies were handsearched for additional relevant studies. A manual internet search for any relevant reports that met the study inclusion criteria was undertaken.

### Eligibility criteria

A broad inclusion criteria was adopted for this review, including grey literature. However, because of resource constraints, it was decided papers not published in English would be excluded. Full details of the inclusion and exclusion criteria are shown in Box 1.

**Box 1.** Inclusion and exclusion criteria

**Inclusion criteria** Studies containing primary qualitative or quantitative data regarding the experiences of young people (age 12–25 years) accessing support for their mental health from general practice. **Exclusion criteria** Studies that did not contain primary data (for example, review articles, opinion pieces, conference abstracts, or case studies). Studies not focused on young people with mental health concerns. Studies that focused only on prospective views of primary care-based mental health care. Studies that involved people outside of the ages of 12–25 years (or data not broken down by age group). Studies not published in English.

### Study selection

**Figure 1.**
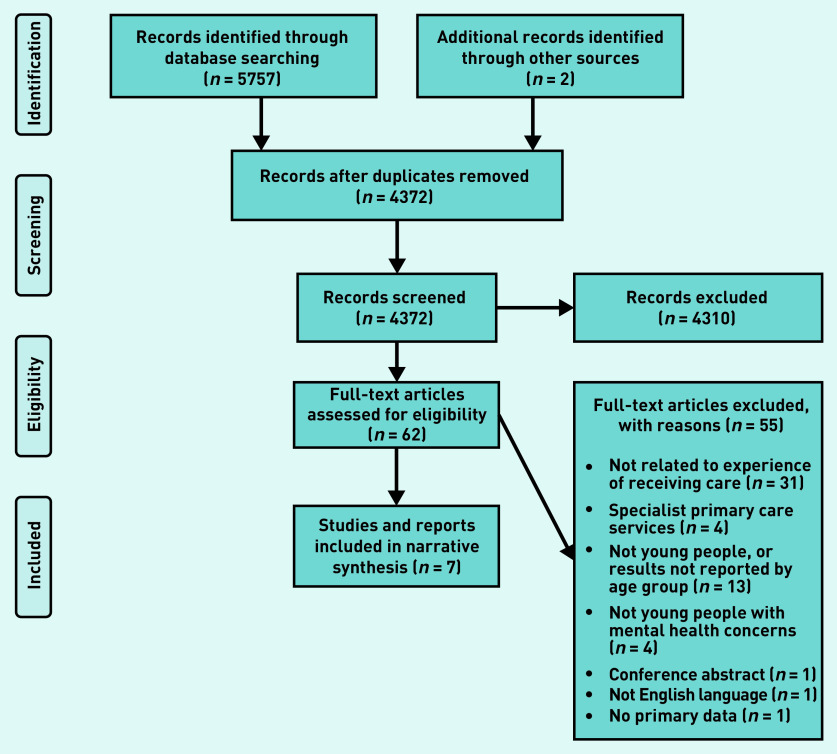
*Prisma flow diagram.*

Search results were imported into a reference management tool (Endnote) for de-duplication before the first stage of screening by title and abstracts. Four authors of this article reviewed the papers: one screened 100% of records, a second screened 50%, and two further authors reviewed 25% each. Any references that met the inclusion criteria, or where there was some uncertainly about eligibility were then screened using their full text. Full-text screening was conducted independently, with the first author screening 100%, and the second and third author screening 50% each. Discrepancies were resolved by consensus through discussion among all reviewers. The numbers of studies included/excluded within each stage of the selection process are outlined in Figure 1.

**Figure 2.**
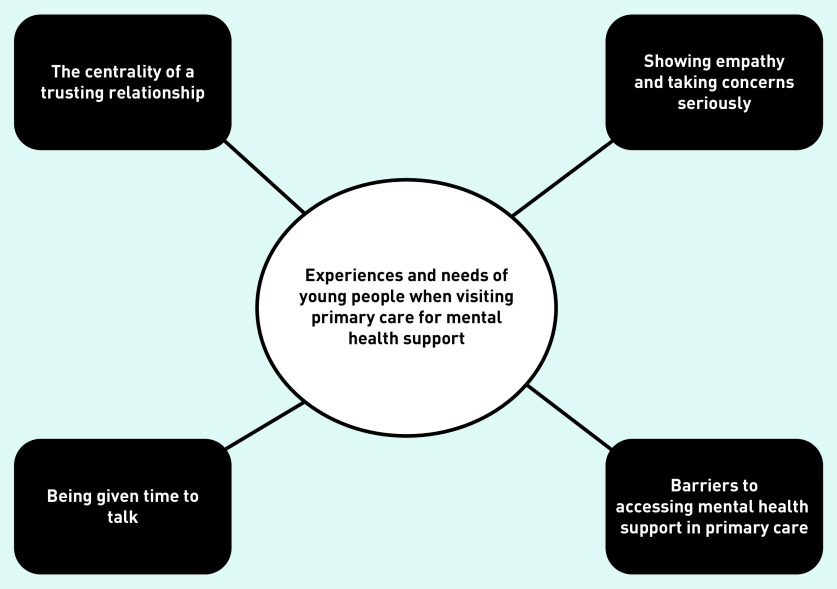
*Overview of themes.*

### Quality assessment

Quality assessment of included studies was conducted independently by two authors using the Mixed Methods Appraisal Tool (MMAT).^12^ Papers were categorised as high, medium, or low quality as specified in Gauly *et al*.^13^ Qualitative and quantitative studies, for which five MMAT criteria exist for each, were grouped into high, medium, or low quality if they met 4–5, 3, or 1–2 criteria, respectively.^13^ For mixed-methods studies, where there are 15 (in one case 20) criteria, papers were divided into high, medium, or low quality if they fulfilled 11–15 (12–20), 8–10 (9–12), or 1–7 (1–8) criteria.^13^

### Data extraction

A data extraction form was created using Microsoft Excel and piloted before independent completion by two reviewers. Extracted data included author, year of publication, country of origin, study aim, population, participant characteristics, study design, mental health concern, or outcomes/evaluation (experiences, perspectives, needs).

### Data synthesis

Elements of the narrative synthesis framework of Popay *et al* ^14^ was used to guide the analysis. This included summarising the characteristics and key findings of included studies, exploring patterns across included studies, and the generation of themes. A narrative synthesis was chosen as this is a common approach to integrate both qualitative and quantitative data.^15^

## RESULTS

A total of five papers^16^^–^^20^ from database searches were included, and two reports from manual internet searching, the YMCA Right Here project report^21^ and a Scottish Youth Parliament report^22^ (Figure 1).

### Study details

The characteristics of included studies are shown in Table 1. The seven studies included involved 1823 young people from four different countries, the UK,^16^^,^^19^^,^^21^^,^^22^ the US,^17^ Ireland^18^ and Canada.^20^ All studies used a qualitative or mixed-methods design and covered a range of mental health problems, including self-harm,^16^ body image and eating concerns,^17^ and psychosis.^19^ Two papers and the reports were not limited to a specific diagnosis and included all young people with mental health or substance misuse concerns.^18^^,^^20^^–^^22^ Two papers also included young people with secondary mental health service use experiences.^19^^,^^20^

**Table 1.** Description of included articles

**Author**	**Year of publication**	**Country of origin**	**Aim**	**Population**	**Study design**	**Research design and methods**	**Quality**
Bailey *et al* ^16^	2019	UK	To explore why young people present to primary care with self-harm and how self-harm consultations in primary care can be improved	Young people with experience of self-harm aged 16–25 years (*n* = 15)	Mixed methods (qualitative data relevant to this review)	Focus groups with young people, GPs, and practice nurses Qualitative data analysed using thematic analysis	High
Kaitz *et al* ^17^	2020	US	To explore women’s barriers to discussing body image concerns with their primary care professionals	Female college students aged 18–35 years (*n* = 102) (results reported by age)	Open-ended questionnaire	Data analysed using the consensual qualitative research method	High
Leahy *et al* ^18^	2018	Ireland	To examine the role of the GP in addressing youth mental health problems	Young people seeking help for mental health and substance misuse problems (*n* = 20) – no specific information on age range, but ‘young people’ was defined as 11–25 years and descriptors associated with quotes included age (range 19–23 years)	Mixed methods (qualitative data relevant to this review)	Semi-structured interviews analysed with thematic analysis	High
Lester *et al* ^19^	2012	England, UK	To explore service user’s perspectives of early intervention services and primary care	Young people with first-episode psychosis aged 18–33 years (*n* = 21) (results reported by age)	Longitudinal qualitative	Semi-structured interviews analysed using a constructivist grounded theory approach	High
Schraeder *et al* ^20^	2017	Canada	To explore the role of the family physician in youth’s mental health care	Young people aged between 12 and 15 years receiving care at children’s mental health services (*n* = 10)	Qualitative interview	Qualitative interviews analysed using constructivist grounded theory	High
Right Here Report; French *et al* ^21^	2011	England, UK	To explore young people’s experiences of visiting their GP and the responses they would like regarding their mental health and wellbeing	Young people aged between 16 and 25 years (*n* = 172)	Mixed methods (qualitative data relevant to this review)	Questionnaires, focus group and interviews	High
Scottish Youth Parliament Report; Burgess *et al* ^22^	2016	Scotland, UK	To explore young people’s views on issues around mental health and accessing services	Young people aged between 12 and 25 years (*n* = 1483)	Mixed methods	Surveys and focus groups with young people Thematic analysis was used to analyse qualitative data and descriptive statistics used to analyse quantitative data	High

### Quality assessment

The methodological quality of all included studies was high. Supplementary Table S2 shows the scores for each of the studies.

### Synthesis of findings

Four themes were generated on the experiences and needs of young people when presenting to primary care for mental health concerns (Figure 2). The themes are outlined below, accompanied by illustrative quotations. The age and sex of quote identifiers have been given where possible, but most sources did not specify this information.

#### The centrality of a trusting relationship

A trusting relationship between the young person and their GP was identified as a key facilitator to accessing mental health support in primary care. Participants reported positive relationships with their GP because of good continuity of care and a long-term patient–doctor relationship. For example, participants emphasised how the trusting relationship between them and their doctor enabled them to access early support for their mental health:

*‘My GP now is cool — she’s awesome. I just sit there and she’s like “so what do you want?”.’ ^21^*

Reassurance from the GP often resulted in positive experiences of the consultation:

*‘Dr. X … told me … “if a pill had* [the capacity to change] *the colour of a person’s eyes differently, you’d be amazed by the amount of different colours of eyes walking past you”, so that was very reassuring to be given that analogy.’ ^18^*

(male, aged 19 years)

Participants also identified the importance of being involved in decisions around their care and being able to discuss treatment options with their doctor. Young people respected doctors who treated them:

*‘… more like an adult than a child.’ ^21^*

#### Showing empathy and taking concerns seriously

Young people reported wanting GPs to listen to their concerns with empathy and make them feel comfortable discussing sensitive information, which facilitated discussions around mental health:

*‘In fact I find it easier to talk to him* [GP] *about my depression than any other health worker I see. That’s because he acknowledges my feelings and he empathises with me.’ ^21^*

In contrast, several participants felt judged and dismissed by their GPs and felt that GPs were insensitive to their concerns about their mental health problems. For example, one participant reported:

*‘I feel that my health care provider doesn’t consider my issues prevalent or pressing issues.’ ^17^*

(aged 22 years)

A key finding across several studies^16^^,^^17^^,^^21^ was the need for GPs to take young people’s concerns seriously during an assessment:

*‘I think they* [clinicians] *can be thinking like…what problems can you have ‘cause you’re, what, 15 or something but no one knows what is happening at home.’* ^16^

#### Being given time to talk

Participants from different studies^16^^,^^17^^,^^20^^,^^21^ reported feeling rushed during GP consultations about mental health concerns. They felt that usual standard consultation time was not long enough to adequately discuss their mental health, either because of the short nature of the consultation or not being given the chance to talk. Young people reported positive experiences when granted a longer consultation to talk about their mental health:

*‘Another thing that really helps is that he is really calm, like he doesn’t hurry you along. If I was to go in there for a couple of things he wouldn’t say “right, you need to hurry up”… he wouldn’t say anything like that, he just lets me be.’*^21^

#### Barriers to accessing mental health support in primary care

Young people across several studies reported not seeking mental health support from their GP because of the perception that GPs should only be consulted for physical health problems:^17^^,^^18^^,^^20^

*‘I don’t think doctors know as much about* [mental health] *. Because they have no experience …’* ^20^

Accessing mental health care through the GP was hampered by previous negative experiences. For example one young person said:

*‘When I sought help from the GP he basically said that… it was just a phase I was going through!’* ^22^

Others felt that their GP did not have a patient-centred approach to care and so did not take their needs into account. In some cases, this was linked to poor communication:

*‘I have had several referrals to CAMHS. The first time the GP didn’t really inform me what CAMHS was. All they said was “I’m going to make this referral” and they spoke into a speaker phone and that was it.’ *^21^

Several studies also mentioned young people found GPs ‘were quick to put a pen to paper’^21^ and prescribe medication, rather than explore other treatment options.

## DISCUSSION

### Summary

To the authors’ knowledge, this is the first review to synthesise evidence on the experiences and needs of young people visiting their general practice for mental health care. Young people with or without a mental health diagnosis or prior mental health service experience were more likely to have a positive experience of sharing their mental health concerns if they had a trusting relationship with their GP, if their GP was empathetic, took their concerns seriously, and took time to listen to them. A lack of these qualities was associated with negative experiences, in addition to the perception that GPs were too quick to prescribe medication. Perceiving that GPs did not know much about mental health problems was an identified barrier for young people engaging with their GP.

### Strengths and limitations

This review was conducted according to PRISMA guidelines,^11^ and screening, selection, extraction, and quality appraisal was undertaken by two independent reviewers. Grey literature was included, which enhanced the richness of findings. Themes were agreed on within a diverse team consisting of different professional backgrounds: social science, psychiatry, psychology, primary care, and applied health services research, which enhances the trustworthiness of findings.^23^

Although the quality of all seven studies included was high, evidence from Western settings only limits the generalisability and transferability of findings to middle- and lower-income primary care settings. With no access to primary data, it was not possible to verify all participant details. For example, it appears that some were receiving additional support alongside input from their GP. This may be a confounding factor in their experiences and/or responses regarding the support they received for their mental health from their GP.

### Comparison with existing literature

The positive experiences young people had during GP consultations in this review are all related to GPs’ ‘relational skills’ that Rocque and Leanza^24^ have identified as being important for patients of any age and with any health problem when communicating with their primary care physician. Empathy and providing time to talk are linked to good listening, understanding, and compassion, which are central in managing people with mental health conditions.^25^ As many young people dislike talking about their feelings, emotions, or thoughts,^26^^,^^27^ empathy and unrushed appointments are even more crucial for this patient group who normally are reluctant in engaging with health care professionals for their mental health.^27^

GPs’ interpersonal and communication skills are also essential for building trust in the GP–patient relationship.^28^ A trusting relationship with the GP can also be built over time through continuity of care, but seeing different GPs may disrupt this, and may result in a breakdown in trust. For young people a trusting relationship is key for discussing more sensitive issues, such as those linked to mental health.^29^ In a recent Australian survey targeting 12- to 24-year-olds, relational continuity with the same GP resulted in better engagement and access, and more positive attitudes to navigating the health system.^30^

The identified barriers for mental health support in primary care are directly associated with some of the key barriers GPs face caring for young people with mental health problems, including lack of time, knowledge, mental health providers, and resources.^31^ Time restrictions directly have an impact on the sensitive task of recognition, diagnosis, and management of problems that is required to avoid referral to stretched specialist services.^31^^,^^32^ Some GPs may be knowledgeable about general mental health conditions, but many feel ill equipped to assess and manage more complex problems in young people, including suicide risk.^33^ Some of the young people in the included studies had prior mental health service experience. In these instances, poor information flow and communication by secondary care may have a negative impact on the care provided by GPs.^34^

‘Recurvisity’ defined by Rogers *et al* describes how future demand for services and the process of help-seeking is determined by a patient’s previous experiences.^35^ In line with this, the current study identified that prior negative experiences of consulting GPs affected young people’s future help-seeking.

### Implications for practice and research

GPs require the ability to maintain continuity of care with young people with mental health concerns, including unhurried consultations. This enables effective interpersonal communication, and the development of rapport and a trusting relationship: a core priority for the Royal College of General Practitioners.^36^ Making sure every contact counts and acknowledging that there is no health without mental health is one way to build rapport and to improve communication about mental health between GPs and young people.

The COVID-19 pandemic has increased the use of remote or virtual online consultations.^37^^,^^38^ There are indications that remote consultation may facilitate engagement in some patient groups.^39^ More research is needed, however, to establish whether young people find it acceptable to consult their GP remotely for their mental health.

Since April 2021, new funding has become available for the option of employing mental health professionals within primary care networks via the NHS Additional Roles Reimbursement Scheme.^40^ Strategic planning of this new mental health practitioner role may support GPs in the management of mental health concerns in young people, closer to home.^1^ Future research needs to explore the perspectives of GPs on supporting young people with mental health concerns, and how they can best manage young people, including those from different cultural and socioeconomic backgrounds within a primary care network context. The incidence rates of young people presenting to primary care for mental health problems over time should be accurately monitored and evaluated.

In conclusion, to conduct high-quality and effective mental health consultations with young people in primary care it is important that young people are given the opportunity to develop a trusting relationship with their GP through unhurried consultations and continuity of care. Various barriers to accessing timely support in primary care need to be overcome to improve young people’s experiences of accessing mental health care.
